# Maximizing solar radiations of PV panels using artificial gorilla troops reinforced by experimental investigations

**DOI:** 10.1038/s41598-024-53873-9

**Published:** 2024-02-12

**Authors:** Ashraf K. Abdelaal, Amira I. A. Alhamahmy, Hossam El Deen Attia, Attia A. El-Fergany

**Affiliations:** 1 Department of Electric Power and Machine, Faculty of Technology, Suez University, Suez, 43512 Egypt; 2 Department of Electric Power and Machine, Faculty of Engineering, Zagazig University, Zagazig, 44519 Egypt

**Keywords:** Optimum tilt angle, Renewable energy, Solar radiations, PV panels, Metaheuristic techniques, Artificial gorilla troops algorithm, Electrical and electronic engineering, Energy harvesting

## Abstract

This article's main objective is to maximize solar radiations (SRs) through the use of the gorilla troop algorithm (GTA) for identifying the optimal tilt angle (OTA) for photovoltaic (PV) panels. This is done in conjunction with an experimental work that consists of three 100 W PV panels tilted at three different tilt angles (TAs). The 28°, 30°, and 50° are the three TAs. The experimental data are collected every day for 181-day and revealed that the TA of 28° is superior to those of 50° and 30°. The GTA calculated the OTA to be 28.445°, which agrees with the experimental results, which show a TA of 28°. The SR of the 28o TA is 59.3% greater than that of the 50° TA and 4.5% higher than that of the 30° TA. Recent methods are used to compare the GTA with the other nine metaheuristics (MHTs)—the genetic algorithm, particle swarm, harmony search, ant colony, cuckoo search, bee colony, fire fly, grey wolf, and coronavirus disease optimizers—in order to figure out the optimal OTA. The OTA is calculated by the majority of the nine MHTs to be 28.445°, which is the same as the GTA and confirms the experimental effort. In only 181-day, the by experimentation it may be documented SR difference between the TAs of 28° and 50° TA is 159.3%. Numerous performance metrics are used to demonstrate the GTA's viability, and it is contrasted with other recent optimizers that are in competition.

## Introduction

Solar energy utilization by PV has become a vital source of renewable energy (RE)^1^. There are many factors affecting the performance of the PV system. The tilt angle (TA) of solar panels is one of these factors which can effectively enhance the electrical energy production. In the last 5 years, numerous researches have been conducted in various countries around the world to determine the OTA for solar panels^2^.

These studies have used various techniques and software to compute the OTA. These include mathematical modeling and numerical simulations, experimental approaches, and Artificial Intelligence (AI)-based methods. In addition, these studies nearly covered most of the world countries such as China, Spain, India, Brazil, Turkey, Algeria, Iraq, Malaysia, Pakistan, Saudi Arabia, Iran, Morocco, Libya, Egypt, Ecuador, Ethiopia, and Uganda. For instance, in Turkey^3^, Cagman found that PV panels tilted at a 30° angle at Bursa city that has latitude angle (LA) of 40.1885° N generates more energy than that is tilted at 13° by 42.17 kWh/year or 2.45%, with CO_2_ production discount of 495 tons for a period of twenty five years. This is agreed with the common rule that suggest the TA is very near to the LA. Oulimar, and Bellaoui^4^ implemented a study to find the OTA in Adrar, Algeria that has LA of 27.874° N. The study was based on collecting data for eleven years. The outcomes of the study stated that the OTA is 28°, which is very near to the LA.

In Ref.^5^, Ponce-Jara and Rus-Casas suggested the use of a tracking system for PV to improve the efficiency of the PV panels in Ecuador. The authors of the current study see that a tracker system is not suitable for PV panels from the practical point of view, since changing the TA repeatedly will add additional costs. A remarkable work was done in Brazil by Cavalcanti and Batista^6^ in which a comparison has been made between three PV systems which were installed in the state of Ceará, Brazil which has LA of -3.731862°. The first and second systems are fixed at TAs of 18° and 22° and the third system has a solar tracker. The total cost of the three systems were 1428.47$, 1428.57$ and 1921.43$ respectively. The system with TA of 18° has the lowest performance, and the system with TA 22° has a performance very near to that with solar tracker but with lesser costs but without the extra cost of the tracker.

Memon and L.G. Hua^7,8^ presented a numerical analysis using MATLAB/Simulink to find the OTA for a 1 MW PV system that was previously installed at 15° TA at Sukkur IBA university in Pakistan that has latitude of 27.73° N. The OTA was found to be 29.5°. Although is OTA is very near to the LA, but the authors of the current study are sure that the OTA is slightly less (not greater) than that of the LA, for example for Suez city that has latitude of 29.95° the OTA is calculated a 28.445°. Xu a and Longin in^9^ investigated the OTA for areas with high elevation in Batang County, Sichuan, China that has latitude 30.0054° N. They used a TA of 40° for best performance. In Ref.^10^, a comparison between two TAs is implemented in Kelantan, Malaysia. The first TA is 40° and the other panel is horizontal. The TA of 40° had better performance than the horizontal surface, which is logical sine the horizontal surface is very far from the LA. A thorough study has been implemented in Ref.^11^ to find the OTA for different cities in Iraq. The study used a data that were collected in 19 years and suggested that the TAs for winter months are different from those for summer months. The study ended by suggesting an annual OTA for 18 cities in Iraq.

In Ref.^12^, a study has been made to find the annual OTA for Yazd city in Iran (Latitude 31.54°) and it was found to be 29.38°, which is convenient since it is slightly less than the LA. Ashetehe in Ref.^13^ computed the seasonal and yearly OTAs for Bahir Dar city (Latitude 11.5742° N) in Ethiopia. Yadav in Ref.^14^ suggested that there is a link between OTA and the LA which is stated by many other authors. Mamun, et al. in Ref.^15^ suggested a 15° is the best TA for all cities in Malaysia (LA 4.2105° N). Similar work was done in Ref.^16^ in Punjab, Pakistan which has LA of 31.1471° N but suggested the same TA of 15^o^ which far from the value of the LA. Karinka and Upadhyaya in Ref.^17^ stated that if the PV panels are not tilted at monthly OTA, there will be an energy loss of 12%. Morad and Al-Sayyab in Ref.^18^ conducted a study to compute the annually and monthly OTA at three cities in Iraq. These cities are the capital, which has LA of 33.33°, Tikrit that has LA of 34.58°, and Diyala, which has LA of 33.233°. The authors suggested that one OTA of 31 for the three cities. Tamoor and Habib in Ref.^19^ implemented a study in Pakistan suggested that a TA of 15° is acceptable for PV panels. Obiwulu and Erusiafe in Ref.^20^ used 6 systems tilted at different TAs in Nigeria, and suggested that a TA of 16.8° gives the best performance. Ashetehe in Ref.^21^ continued his work made in Ref.^13^ and suggested OTA for some cities in Ethiopia. The OTAs were computed in some cities in SA in by Mansour in Ref.^22^ and he suggested that for the city of Riyadh that has LA of 24.7136° N, a TA of 22.7 was chosen to be the best TA.

Farahat in Ref.^23^ selected TAs of 30°, 20° and 25° for some cities in SA. Sharma in Ref.^24^ suggested the monthly OTA for Pradesh, India that has LA of 31.7° N. Sahin in Ref.^25^ used ANN to compute the OTA in Turkey and stated that the OTA can rise SR by 34%. Taha and Hameed^26^ computed the OTA in Duhok University that has LA of 36.862° and its value was 32.7°. Nfaoui in Ref.^27^ computed the OTA that gives the optimal SR in Morocco. Abdelaal, et al. in Ref.^28^ conducted through simulations to determine the OTA for each day, each month, each season, and for the whole year. They computed the OTA in some cities in EGYPT. Celik in Ref.^29^ used ANN to predict SR. H. Hussein in^30^ discussed the effect of varying TA on SR. H. Khorasanizadeh in Ref.^31^ tried to find the OTA in Iran. S. Oliveira-Pinto in Ref.^32^ analyzed the new merging devices in the calculation of SR. M. A. Ramli in Ref.^33^ treed to use ANN to estimate SR. S. P. Simon in^34^ investigated the effect of economic aspects on the PV systems. K. Skeiker in Ref.^35^ estimated the OTA in Syria. S. Soulayman in Ref.^36^ computed the OTA for building. C. Stanciu in Ref.^37^ estimated the OTA and presented 3 techniques to compute SR. R. Xu in Ref.^38^ investigated the OTA on SR. R. Yan in Ref.^39^ estimated the OTA in Australia. A. Barbón in^40^ tried to give a general model to enhance the PV panel. E. González-González in Ref.^41^ tested the effect of TA on SR in the Iberian Peninsula.

Recently heuristics methods have been used to get the OTA. For example in Ref.^42^, the particle swarm (PS) and Bee colony (BC) were used to calculate the OTA. The results shows that a very slight difference between the two methods. Most of the pervious methods showed that the OTA is very near to the LA and the difference between the two computed angles in the range of 1 to 3 degrees.

Since TA has great effects on SR, this work is focused on estimating the OTA by GTA, beside with an experimental work on three PV tilted systems. In addition, other nine MHTs were used to compare the results with that obtained from GTA.

The following are the important contributions of this work: (i) first, to illustrate the relevance of the TA that provides practically consistent SR throughout the year; otherwise, money will be lost. (ii) Using GTA and the other nine MHTs to compute the OTA, and. (iii) The results are supported by experimental work performed with three PV panels at three distinct tilt angles.

This paper is sorted as follow; the first section of the paper displays the previous work done in the computation of the OTA. Section "Mathematical model of SR" reviews the basic relations of SR on both horizontal and tilted surfaces. Section "The effect of the TA in the extra-terrestrial solar radiation (SR)" analyzes the importance of the correct TA that gives a nearly constant SR all the year. Section "Metaheuristic techniques (MHTs)" investigates the application of the GTA beside with 9 different MHTs to compute OTA. The 9 MHTs are genetic algorithm (GA), PS, harmony search (HS), ant colony (AC), cuckoo search (CS), BC, fire fly (FF), grey wolf (GW), and coronavirus disease optimizer (COVIDO). Section “Simulation results and discussions” shows the simulation results of these different techniques. The performance measures and analysis are made available in Section "Performance analysis". The verifications through experimental setup are revealed in Section "Experimental work". The last Section "Conclusions" presents the experimental work that are implemented on 3 different TAs.

## Mathematical model of SR

The Mathematical model of SR can be described by the following equations^43^:

First the square of the inverse of the distance between sun and earth can be calculated from

1 $${{\text{E}}}_{o}={\left(\frac{{{\text{d}}}_{o}}{{\text{d}}}\right)}^{2}=1.00011+3.4221.1{0}^{-2}\mathrm{cos\epsilon }+1.28.1{0}^{-3}\mathrm{sin \epsilon }+7.19.1{0}^{-4}\mathrm{cos }2\upepsilon +7.7*1{0}^{-5}{\text{sin}}2\in$$

where $\upepsilon$ is the angle of the day calculated from the following equation:

2 $$\epsilon =2\pi \left(n-1\right)/365$$

The declination angle δ can be calculated from

3 $$\updelta =\left(\mathrm{6.918.1}{0}^{-3}-0.399912\mathrm{cos\epsilon }+7.0257.1{0}^{-2}\mathrm{sin\epsilon }-6.758.1{0}^{-3}{\text{cos}}2\upepsilon + 9.07.1{0}^{-4}{\text{sin}}2\upepsilon - 2.697.1{0}^{-3}{\text{cos}}3\upepsilon + 1.48.1{0}^{-3}{\text{sin}}3\upepsilon \right).\left(\frac{180}{\pi }\right)$$

The angle of the sun set with respect to a horizontal surface ${\upomega }_{{\text{s}}}$ is calculated as

4 $${\upomega }_{{\text{s}}}={{\text{cos}}}^{-1} (-\mathrm{tan\varphi }.\mathrm{tan\delta })\mathrm{ in \,degrees}$$

where the angle φ is the latitude angle. The sun set hour angle of an inclined surface ${\upomega }_{{\text{s}}}^{\mathrm{^{\prime}}}$ at a TA β is calculated as

5 $${\upomega }_{{\text{s}}}^{\mathrm{^{\prime}}}=\mathrm{min }\left\{{\upomega }_{{\text{s}}},\mathrm{ co}{{\text{s}}}^{-1} \left[-\mathrm{tan \delta tan }\left(\mathrm{\varphi }-\upbeta \right)\right]\right\}$$

The SR at horizontal surface H_o_ is

6 $${{\text{H}}}_{{\text{o}}}=\frac{24}{\uppi }.{{\text{I}}}_{{\text{SC}}}.{{\text{E}}}_{{\text{o}}}.\mathrm{cos\delta }.\mathrm{ cos\varphi }.[{\text{sin}}{\upomega }_{{\text{s}}}-(\uppi /180){\upomega }_{{\text{s}}}{\text{cos}}{\upomega }_{{\text{s}}}]$$

While The SR on inclined surface H_oβ_ is

7 $${{\text{H}}}_{\mathrm{o\beta }}=\frac{24}{\uppi }.SC.{{\text{E}}}_{{\text{o}}}\left[ \frac{\uppi }{180} {\upomega }_{{\text{s}}}^{\mathrm{^{\prime}}}\mathrm{sin \delta }.{\text{sin}}\left(\mathrm{\varphi }-\upbeta \right)+\mathrm{cos\delta }.{\text{cos}}\left(\mathrm{\varphi }-\upbeta \right).{\text{sin}}{\upomega }_{{\text{s}}}^{\mathrm{^{\prime}}}\right]$$

where SC is the solar constant. Equations (1), (2), (3), (4), (5), (6) and (7) represent the complete system for estimating the SR at horizontal surface or inclined surface at an angle β.

The terrestrial solar radiation (TSR) depends on area characteristics. In general, TSR is divided into direct, diffused, and reflected radiation. There are many formulas to obtain the TSR. For example, Angstrom^61^ suggested a formula that relate TSR to extraterrestrial SR, which is given by

8 $$\frac{H}{{H}_{o}} =a+b\left(\frac{n}{N}\right)$$

where $H$ is the TSR (MJ/m^2^/day), H_o_ is th extraterrestrial SR, n is the daily sunshine hours (hr), N is the maximum daily hours. Bakirci K in Ref.^61^ presented another equation to relate terrestrial and extraterrestrial SR in the formula given in Eq. (9).

9 $$\frac{H}{{H}_{o}} =a {\left(\frac{n}{\overline{N}}\right)}^{b}$$

where *a* and *b* are constants have to be determined locally. For simplicity in this paper consider the second equation with a = b = 1. This is acceptable in Suez area, since it has clear and sunny sky almost the days of the year.

## The effect of the TA in the extra-terrestrial solar radiation (SR)

**Figure 1:**
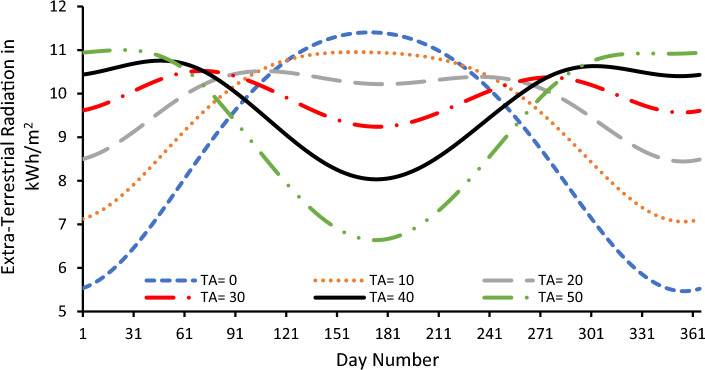
Effect of the TA in the SR when the TA is away from the TA.

In order to show the effects of the TA on the SR, Eqs. (1), (2), (3), (4), (5), (6) and (7) are simulated using MATLAB for different TAs and their SR are shown in Fig. 1. The blue curve is drawn for a TA of 30° which is very close to the LA of the Suez city. This curve shows that the SR varies slowly all over the year and the maximum value of SR is 10.5696 kWh/m^2^/day and the minimum value is 9.2192 kWh/m^2^/day difference between maximum and minimum values is 1.3504 kWh/m^2^/day or 14.65%.

As the TA goes away from the LA, the difference between the maximum and minimum becomes higher. For example for a TA of 50°, the maximum value of the SR is 11.765 kWh/m^2^/day while the minimum value is 6.5941 kWh/m^2^/day. The difference between maximum and minimum values in this case is 5.1708 kWh/m^2^/day or 78.4158% of the minimum value which is too high and not acceptable from costs point of view since this can leads to waste money during the days of low SR. On the other hand if the TA is zero as for a horizontal surface, the maximum value of the SR is 11.4243 kWh/m^2^/day and the minimum value is 5.5511 kWh/m^2^/day. With a difference between the two values of 5.1708 kWh/m^2^/day or 105.8% of the minimum value which is also not acceptable for economic reasons as mentioned before. So one can state that when the TA is near the LA, the difference between the minimum and maximum value of the SR curve becomes smaller as shown from Fig. 1. When the TA departs away from the LA the difference between the minimum and maximum value of the SR curve becomes higher as shown from Fig. 1 and money waste occurs at these TAs.

**Figure 2:**
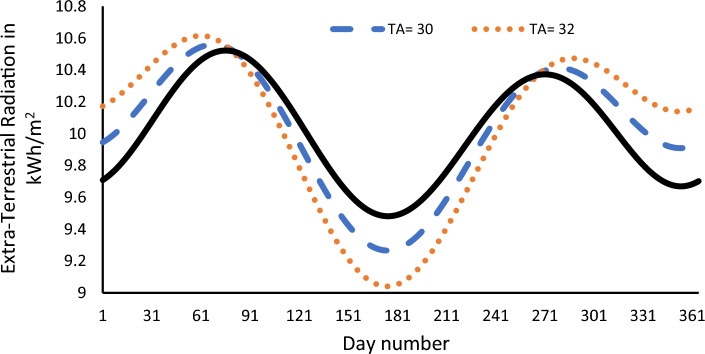
Effect of the TA in the SR when the TA is close to the TA.

Figure 2 shows the SR for 3 close TAs, which are 28°, 30°, and 32°. As shown from the figure, as we go above the LA, the characteristics of the SR diverge, and the difference between maximum and minimum values increases. Vice versa as the TA becomes close from the LA, the difference between maximum and minimum angles decreases. As we stated before the best TA has minimum value between maximum and minimum value. According to this the best TA in Fig. 2 is equal to 28°.

## Metaheuristic techniques (MHTs)

MHTs have appeared as great optimization methods^44^. Currently there are more than 500 MHTs are used in optimization^48^. Since MHTs are characterized by flexibility, adaptively and have excessive search capability, they found wide spread in different applications.

Although GA was the first MH method introduced by Holland^47^, GA is still the most used optimization method as mentioned in a survey in Ref.^50^. A good review on GA is presented in Ref.^49^. There are three important parameters affecting the operation of GA. These parameters are crossover probability, mutation probability and size of the population. The GA is a simple, requires less data and efficient technique for solving optimization problems^49^. The PS^46^ is developed and because of its simplicity, it is considered as the second wide spread used method in optimization^48^. The parameters used in PS are the particles location and their velocity. PS needs a smaller parameters to be tuned and has good convergence. The HS method has less computation efforts and is easy to implement^51^. The key parameters affecting the operation of HS are distance of the bandwidth, size of memory; rate of pitch adjustment, and choosing from memory rate. AC and BC are MHTs which are inspired by the social behavior of both ants^45^ and bees^52^. The main parameters used in AC are the evaporation and weighting of pheromone. In CS^53^, there are lesser parameters to be tuned and based on levy flight. CS can be very efficient in finding global solution. FF^54^ was inspired by the blinking process of fireflies and is used wildly in different applications. The main parameter in the FF algorithm is the brightness of FF. Recently, the GW^55,56^ which imitates the hunting process of gray wolves. Recently the GTA^57^ got a wide interest in many applications because of its superior convergence and it will be discussed in more details in the next section. The COVIDO^62^ is a simple and new algorithm and will be used to find the OTA.

### Artificial gorilla troops algorithm

Like any society, Gorillas have sensitive state, have solid family ties, hunt for their own food and have a view about their past and future. Their foods fluctuate from one gorilla type to other types. Gorillas live in highlands, are mostly vegetarian. The leader of the group is called Silverback (SB) and he is the pivot of a group and he is in charge of many responsibilities like leading, searching and protecting of the group individuals. A good review about social behavior of GTA can be found in^57^.

### Procedures of the GTA

There are two phases that mimic the GTA behavior. The first phase is called exploration phase (EXP), and the second phase is the exploitations phase (EXTP). In the EXP, there are three places that a gorilla could migrate to it according to Eq. (10). The first one is to go unknown place when the migration probability parameter *p* is greater than a random number, the second is to go to known place when the random number is greater than or equal to 0.5. The third one is to go to other gorilla when the random number is less than 0.5. The parameter p should be specified at the beginning of the optimization process.

10 $$GX\left(IT+1\right)=\left\{\begin{array}{ll}\left(Upp\_B-L\_B\right)*{r}_{1}+L\_B & \quad rand < p\\ \left({r}_{2}-C\right)\times {X}_{r}\left(IT\right)+L\times H & \quad rand\ge 0.5\\ X(k)-L\times \left(L\times \left(X\left(IT\right)-G{X}_{r}\left(IT\right)\right)+{r}_{3}\times \left(X\left(IT\right)-G{X}_{r}\left(IT\right)\right)\right) & \quad rand < 0.5\end{array}\right.$$

In Eq. (8), *Upp_B* is the upper bound of the designing variables, and *L_B* is the lower bound and *rand*, *r*_*1*_, *r*_*2*_*, r*_*3*_ and *rand* are random numbers. The value of C, L and H can be determined by Eqs. (11), (12), (13), (14) and (15). *X* denotes the position of the gorilla, ${X}_{r}$ is a randomly chosen gorilla, *X(IT)* is the position of the gorilla at iteration number IT, *X(k) gorilla number k, and* GX(IT + 1) is the new position of the gorilla at iteration (IT + 1).

11 $$C=F\times \left(1-\frac{IT}{Max\_IT}\right)$$

12 $$F={\text{cos}}\left(2\times {r}_{4}\right)+1$$

13 $$L=C\times l$$

14 $$H=Y*X(IT)$$

where F, L, and H are internal variables used to simulate the SB gorilla’s leadership, *r*_*4*_ and *l* are random numbers and *Y* is a random number limited between—C to C or

15 $$Y = [-C, C]$$

On the other hand, there are two processes in the EXTP. The first one is to follow the SB when *C* ≥ *W*. These can be described by Eqs. (16), (17), (18).

16 $$GX\left(IT+1\right)=L*M*\left[X\left(IT\right)- {X}_{silverback}\right]+X(IT)$$

where L is calculated as in Eq. (12) and M is given as

17 $$M= {\left[{\left|\frac{1}{{N}_{pop}} \sum_{j=1}^{{N}_{pop}}G{X}_{j}(IT)\right|}^{g}\right]}^{(\frac{1}{g})}$$

18 $$g= {2}^{L}$$

where ${{\text{X}}}_{{\text{silverback}}}$ is the SB position.

The other process is to contest for adult female when *C* < *W*, which can be described by Eqs. (19), (20), (21) and (22).

19 $$GX\left(k\right)={X}_{silverback}-\left[ {X}_{silverback}*Q-X\left(IT\right)*Q\right]+X(IT)]*A$$

20 $$Q=2* {r}_{5}-1$$

21 $$A= \beta *E$$

22 $$E= \left\{\begin{array}{ll}{N}_{1} & \quad rand \ge 0.5 \\ {N}_{2} & \quad rand<0.5\end{array}\right.$$

**Figure 3:**
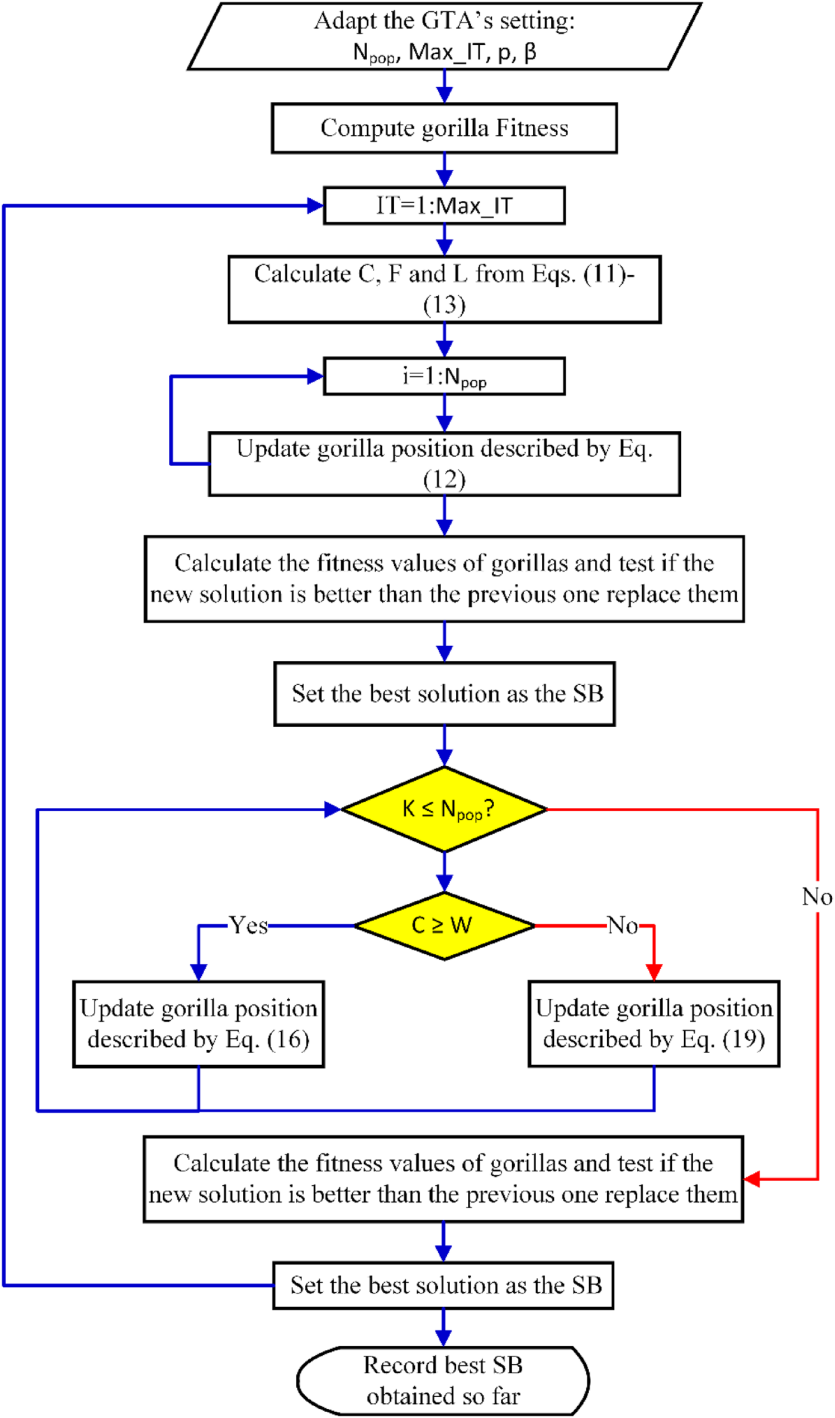
Implementation of the GTA.

The complete flow chart showing the GTA is shown in Fig. 3.

The GTA algorithm is implemented using MATLAB to find the OTA, and the results are investigated in the next portion.

### Determination of the OTA by using the GTA

The key objective is to find the OTA for PV panels that gives the maximum SR. The optimization problem can be written with the objective function (OF) which is adapted to maximize the SR on a tilted surface by an angle λ, described by:

23 $${{\text{H}}}_{\mathrm{o\beta }}=\frac{24}{\uppi }*SC*{{\text{E}}}_{{\text{o}}}\left[ \frac{\uppi }{180} {\upomega }_{{\text{s}}}^{\mathrm{^{\prime}}}\mathrm{sin \delta }.{\text{sin}}\left(\mathrm{\varphi }-\upbeta \right)+\mathrm{cos\delta }.{\text{cos}}\left(\mathrm{\varphi }-\upbeta \right).{\text{sin}}{\upomega }_{{\text{s}}}^{\mathrm{^{\prime}}}\right]$$

The SR is subjected to the constraints depicted in Eqs. (1), (2), (3), (4), (5) and (6).

All variable names and their definitions are mentioned in Section "Mathematical model of SR". The objective is to use GTA to solve the OTA problem defined by Eq. (23).

## Simulation results and discussions

The previous optimization problem has been simulated in MATLAB using the GTA. The different parameters in GTA used in simulation are given as follows: Population Size, ${N}_{pop}$ = 30, maximum number of iteration, Max_IT = 50, number of designing variables = 1 with L_B =${0}^{o}$ and UPP_B = ${90}^{o}$ and The migration probability parameters with p = 0.03, β = 3 and W = 0.8. It may be good to mention that these adapted settings are cropped using trails and errors methodology to have better performance of the employed optimizer. Table 1 announces the key parameters of the 9 previously mentioned challenging methods.

**Table 1:** The adapted control parameters of the 9 MHTs,

Method	Main Parameters	Method	Main Parameters
GA	Number of population = 50 Number of generations = 20 Number of elitism children = 2	CS	Number of nests = 100 Discovery rate of alien eggs = 0.25 Levy exponent and coefficient = beta = 3/2;
PS	Swarm Size = 50 Inertia Weight = 1 Inertia Weight Damping Ratio = 0.99 Personal Learning Coefficient = 1.5 Global Learning Coefficient = 2	BC	Population Size = 100 Number of Onlooker Bees = 100 Acceleration Coefficient Upper Bound = 1
HS	Harmony Memory Size = 50 Number of New Harmonies = 20 Harmony Memory Consideration Rate = 0.9 Pitch Adjustment Rate = 0.1 Fret Width Damp Ratio = 0.995	FF	Number of Fireflies = 100 Light Absorption Coefficient = 1 Attraction Coefficient Base Value = 2 Mutation Coefficient = 0.2 Mutation Coefficient Damping Ratio = 0.98
AC	Number of Ants = 100 Pheromone Exponential Weight = 0.3 Evaporation Rate = 0.1 number of divisions = 90	GW	Number of search agents = 24 The parameter a The coefficient vectors A and C
COVIDO	Population Number = 100 Mutation Rate = 0.1 Number of Proteins = 2 Shift number = 1		

**Figure 4:**
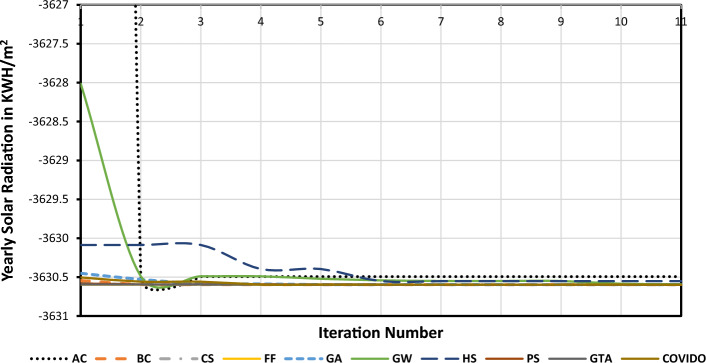
The convergence patterns of the MHTs.

The convergence trend of the GTA is shown in Fig. 4 which shows the values of the OF (multiplied by − 1 for maximization purpose) versus the iteration number. The AC and HS have the worst convergence process at the first few iterations.

**Figure 5:**
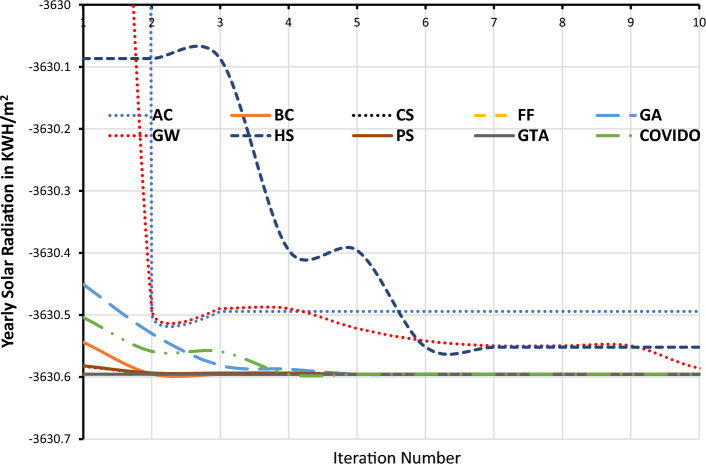
A clearer view of the convergence patterns of the MHTs.

The remaining MHTs including GTA have similar convergence characteristics. In order to discriminate between these remaining MHTs, Fig. 5 shows a clearer view of the convergence process. The horizontal black line represent 6 MHTs that have nearly same characteristics, and cannot differentiate between them. The 6 MHTs are BC, FF, CS, PS, GW, and GTA. The OTA for some cities around the world are obtained from these MHTs and are shown in Table 3. Also the SR in kWh/m^2^/year is calculated for each OTA. The results are shown in Table 3 which shows the OTA and SR obtained by each methods.

The OTA of Suez city was calculated in Ref.^28^ to be 28° and was calculated in the previous section by GTA as 28.445° but the MHTs identify the OTA to be 28.446°. A slightly different solution is found by AC which gives an OTA of 28.507° and yearly SR of 3630.6 kWh/m^2^ which is nearly equal to the SR calculated from other methods. For the sake of comparison the OTAs for some other cities in other countries are calculated. For example the OTA for Madinah city in SA is reported in Ref.^58^ as 23.5°, but as shown in Table 1 by using MH methods, the OTA of Madinah is 23.3° which gives a SR of 3640 kWh/m^2^/year. For the Mecca city, the OTA is calculated as 20.34°. In Ref.^59^, the OTA of Doha, Qatar, was estimated to be 25°, but using MH methods give OTA of 24°. For Dubai city, the OTA was calculated in Ref.^60^ as 23.39° but using MH algorithms the corrected OTA of Dubai city is 23.9 ^o^ which gives better SR. Table 2 shows the OTA of some cities in different countries including Suez city in Egypt calculated using GTA and the other 9 methods.

**Table 2:** Yearly SR in kWh/m^2^/year at OTA.

Method	Suez LA 29.9989° N		Mecca (SA) LA 21.3891° N		Madinah (SA) LA 24.5247° N		Doha (Qatar) LA 25.2854° N		Dubai (UEA) LA 25.2048° N	
	SR	OTA	SR	OTA	SR	OTA	SR	OTA	SR	OTA
GA	3630.596	28.446	3644.6	20.34	3640.4	23.3	3639.2	24.0	3639.3	23.9
PS	3630.596	28.445	3644.6	20.34	3640.4	23.3	3639.2	24.0	3639.3	23.9
HS	3630.600	28.446	3644.6	20.18	3640.4	23.2	3639.2	24.0	3639.3	23.8
AC	3630.600	28.507	3643.3	18.76	3638.7	21.5	3638.4	25.3	3638.4	25.3
BC	3630.596	28.445	3644.6	20.35	3640.4	23.3	3639.2	24.0	3639.3	23.9
FF	3630.596	28.12	3644.6	20.69	3640.4	23.8	3639.2	23.6	3639.3	24.1
CS	3630.596	28.445	3644.6	20.14	3640.4	23.3	3639.1	23.6	3639.3	23.9
GW	3630.596	28.445	3644.6	20.34	3640.4	23.3	3639.2	24.0	3639.3	23.9
GTA	3630.596	28.445	3644.6	20.34	3640.4	23.3	3639.2	24.0	3639.3	23.9
COVIDO	3630.596	28.451	3644.6	20.45	3640.36	23.27	3639.2	24.1	3639.3	23.976

## Performance analysis

In order to compare the results obtained from all the nine methods, there many aspect can be taken into consideration but here we will consider firstly the accurate results, Technique complexity, CPU processing time, and also a parametric test will be performed on these MHTs. The independent runs are implemented on a PC with an Intel i7 4970 CPU processer running at 3.6 GHz. It is clear that the GTA, has the best accurate results, and the AC has the worst results this is clear from Fig. 5. Other method are close to the GTA, but the GTA is the best.

### CPU processing time

To compare the different optimizers in terms of CPU processing time, each implemented approach is performed and the computation time is recorded; the results are displayed in Table 3. Table 3 shows that the GTA has the shortest CPU processing time (3.506 s), while the AC has the longest.

**Table 3:** CPU processing time.

Method	GTA	GW	AC	HS	BC	FF	PS	CS	GA	COVIDO
CPU Time (Sec.)	3.506	3.981	1073.6	7.223	9.573	225.28	5.075	8.243	13.888	9.630949

### Parametric tests

Parametric tests are statistical tests that suggest the data nearly tracks a normal distribution. Parametric test is a statistical test that assume the data nearly follows a normal distribution curve. To implement and determine the p-value for each metaheuristic technique, the authors wrote MATLAB code. Mathematically, the p-value is the probability of observing the data obtained inconsistent with the null hypothesis, under the assumption that the null hypothesis is true. A p-value is used to support or reject the null hypothesis. The null hypothesis is usually the idea that two or more groups being compared are identical. The lesser the p-value, the robust the evidence to reject the null hypothesis. If the p-value is less than some predetermined number, such as 5% level of confidence, then the null hypothesis is rejected in favor of an alternative hypothesis, usually that the groups are different by some predefined amount. The Shapiro–Wilk Test is used to compute the p-value. The following steps represents the elementary approach used in the Shapiro–Wilk test for normality:

- Arrange the data in ascending order so that ${x}_{1} \le \dots \le {x}_{n}.$
- Calculate SS as follows:
  24 $$SS= {\sum }_{j=1}^{n}({x}_{j}- \overline{x }{)}^{2}$$
- If n is even, let m = n/2, while if n is odd let m = (n–1)/2
- Calculate b as follows, taking the a_i_ weights from the Shapiro–Wilk Tables.
  25 $$b= {\sum }_{j=1}^{m}{a}_{j}({x}_{n+1-j}- {x}_{j})$$
- Calculate the test statistic: W = b^2^⁄SS.

From the Shapiro–Wilk Tables (for a given value of n) find the closest value to W, interpolating if necessary. This is the p-value for the test. In order to perform the parametric test, each method is implemented for 20 times, and each time the OTA is recorded. The results are shown in Table 4. Although all methods have a very small p-value As shown from the last raw and the GTA has the lowest p-value.

**Table 4:** The values of the OTA against run number for each method.

Run	GTA	GW	AC	HS	BC	FF	PS	COVIDO
1	28.4450	28.4453	28.0068	28.4413	28.4453	28.4651	28.4450	28.4644
2	28.4450	28.444	28.0069	28.4444	28.4419	28.3966	28.4450	28.4409
3	28.4427	28.4451	28.0069	28.4441	28.4437	28.4424	28.4450	28.4603
4	28.4450	28.446	28.0067	28.4461	28.4444	28.3747	28.4450	28.3899
5	28.4447	28.445	28.0069	28.4451	28.4450	28.4157	28.4447	28.4046
6	28.4452	28.4481	28.0069	28.4461	28.4449	28.4424	28.4452	28.5330
7	28.4446	28.4447	28.5068	28.4554	28.4458	28.3747	28.4414	28.4666
8	28.4450	28.445	28.5068	28.4436	28.4450	8.4221	28.4450	28.4076
9	28.4451	28.4446	28.0069	28.4451	28.4453	28.4328	28.4450	28.4470
10	28.4450	28.4449	28.0069	28.4421	28.4450	28.4451	28.4451	28.4379
The P-value	1.385*10^−5^	0.0069	1.267*10^−4^	0.0041	0.0098	1.456*10^−5^	3.864*10^−5^	0.0021

The p-value of the GTA is the lowest while the BC is the highest, although all methods have p-value that is lesser than 0.05.

*GTA* gorilla troops; *PSO* Particle Swarm; *BC* Bee colony; *GA* genetic algorithms; *HS* Harmony Search; *AC* Ant colony; *CS* Cuckoo Search; *CS* Cuckoo Search; *FF* Fire Fly; *GW* Grey Wolf; *COVIDO* coronavirus disease optimizer; *p*-value value of parametric test.

## Experimental work

**Figure 6:**
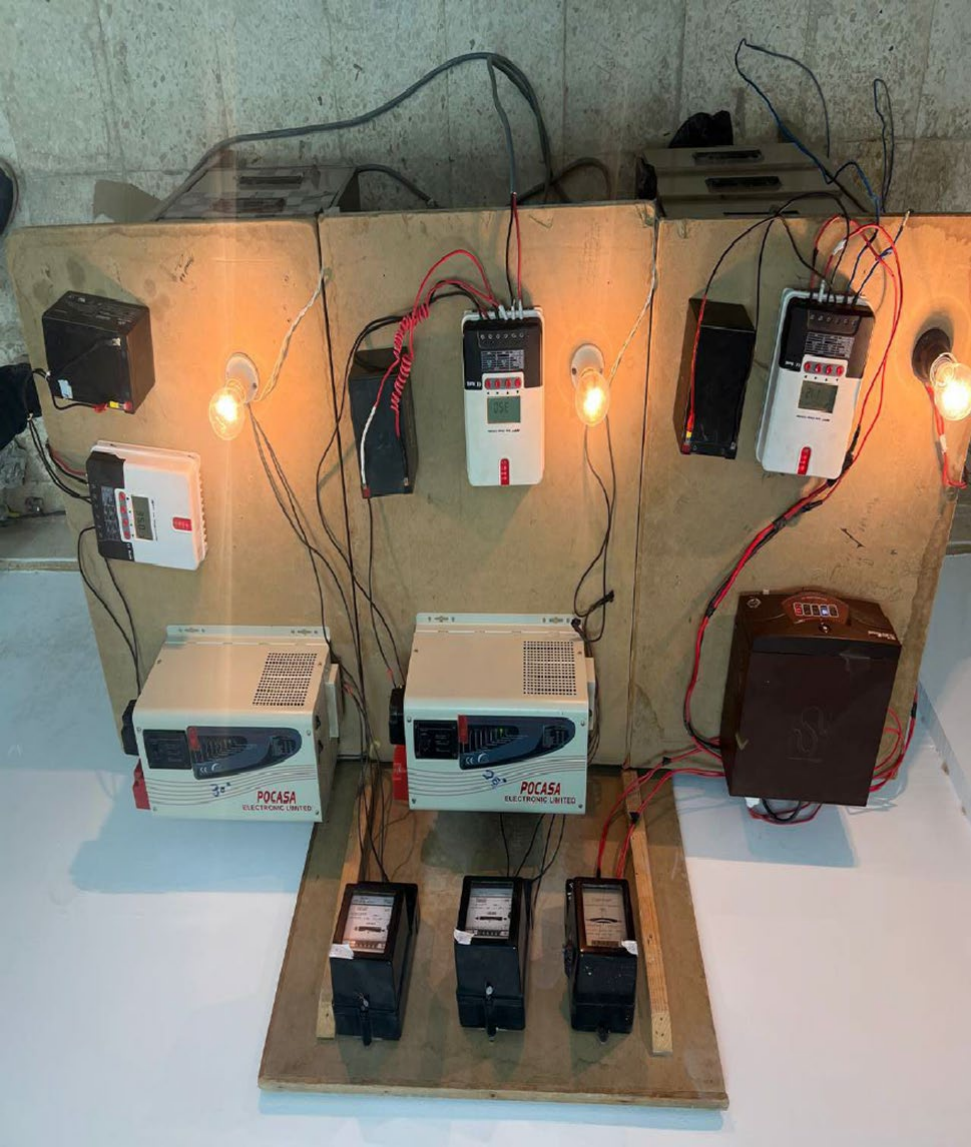
The experimental installed setup.

**Figure 7:**
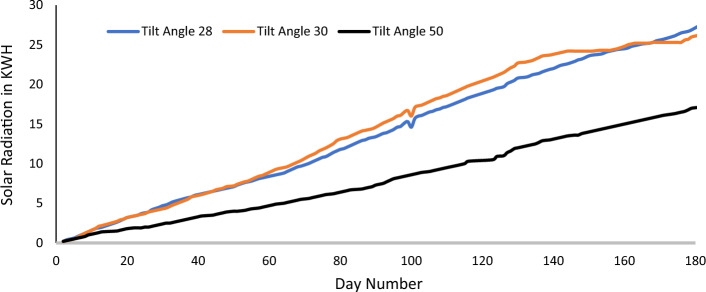
The SR for the experimental three different TAs in kWh.

To verify the theoretical work, an experimental work consists of three PV panels were assembled at Suez University, that has latitude of 30°. The PV panels were directed towards the south and has different TAs of 28°, 30° and 50°. Each PV system consists of one hundred watts PV panel, a peak power point tracker rated 500 W, 1000 W DC-AC converter, 50 W storage battery, and 60 W electrical load. The components are conceded as shown in Fig. 6. There are two panels from these were used last year in^28^, but the measurements were reported only for 60-day. In the present work, measurements were recorder for a period of 181-day, starting from Saturday 24th of December 2022, and ending on Saturday 24th June 2023. The measurements are shown in Table 5. The data is given in Table 2 and is drawn in Fig. 7. From this figure, one can see that the TA of 28° has the best performance. The SR of TA 28° is better than the SR of TA 50° by 59.3% and better than the TA of 30° by 4.5%. It should be noted that these results not for all the year but only for 181-day.

**Table 5:** SR for the different experimental TAs in kWh.

Day	TA			Day	TA			Day	TA			Day	TA		
	28^o^	30^o^	50^o^		28^o^	30^o^	50^o^		28^o^	30^o^	50^o^		28^o^	30^o^	50^o^
1	0	0	0.2	48	6.9	7.1	4.0	93	13.9	15.3	7.9	143	22.5	24.1	13.5
2	0.2	0.2	0.3	50	7.1	7.2	4	94	14.1	15.5	8.1	144	22.6	24.2	13.6
3	0.4	0.3	0.4	51	7.3	7.4	4.1	95	14.3	15.7	8.2	146	22.9	24.2	13.6
4	0.5	0.4	0.5	53	7.6	7.7	4.2	96	14.6	16.0	8.3	147	23.1	24.2	13.8
5	0.6	0.5	0.6	54	7.7	7.8	4.3	97	14.7	16.1	8.4	148	23.2	24.2	13.9
6	0.8	0.7	0.8	55	7.8	8.0	4.4	98	15.1	16.5	8.5	149	23.4	24.2	14.0
8	1.2	1.2	1.0	57	8.1	8.4	4.5	99	15.3	16.7	8.6	150	23.6	24.2	14.1
9	1.4	1.4	1.1	58	8.2	8.5	4.6	100	14.6	16.0	8.7	151	23.7	24.2	14.3
10	1.6	1.6	1.2	59	8.3	8.7	4.7	101	15.7	17.1	8.8	153	23.8	24.3	14.4
11	1.8	1.8	1.3	60	8.4	8.9	4.8	102	16.0	17.3	8.9	154	23.9	24.3	14.5
12	1.9	2.1	1.4	61	8.5	9.1	4.9	103	16.1	17.4	9.0	155	24.1	24.3	14.6
13	2.0	2.2	1.5	62	8.6	9.3	5.0	105	16.5	17.8	9.1	156	24.2	24.3	14.7
17	2.6	2.7	1.6	64	8.8	9.5	5.1	106	16.6	18.0	9.2	157	24.3	24.4	14.8
18	2.8	2.9	1.7	65	9.0	9.6	5.2	107	16.8	18.2	9.3	158	24.4	24.5	15.0
19	3.0	3.0	1.8	66	9.2	9.8	5.3	108	16.9	18.3	9.4	160	24.5	24.8	15.1
20	3.2	3.2	1.9	67	9.4	10.0	5.4	109	17.1	18.5	9.5	161	24.6	25.0	15.2
22	3.4	3.4	1.9	68	9.6	10.2	5.5	110	17.2	18.6	10.0	162	24.8	25.1	15.3
23	3.5	3.5	1.9	69	9.7	10.4	5.6	115	18.1	19.6	10.3	157	24.3	24.4	14.8
24	3.7	3.6	2.0	71	10.0	10.9	5.7	116	18.3	19.8	10.5	158	24.4	24.5	15.0
25	3.8	3.7	2.0	72	10.2	11.1	5.8	123	19.3	20.9	10.9	160	24.5	24.8	15.1
26	3.9	3.9	2.1	73	10.4	11.3	5.9	124	19.5	21.1	11.0	161	24.6	25.0	15.2
27	4.2	4.0	2.3	74	10.6	11.6	6.0	126	19.7	21.5	11.4	163	24.9	25.2	15.4
29	4.5	4.2	2.4	75	10.8	11.8	6.1	127	20.1	21.8	11.6	164	25.0	25.2	15.5
30	4.7	4.3	2.5	76	10.9	12.0	6.2	128	20.3	22.1	11.9	165	25.1	25.2	15.7
31	4.8	4.4	2.5	78	11.4	12.5	6.3	129	20.5	22.3	12.0	167	25.2	25.2	15.8
32	5.0	4.6	2.6	79	11.6	12.9	6.4	130	20.8	22.7	12.2	168	25.3	25.3	15.9
33	5.2	4.8	3.0	80	11.8	13.1	6.5	132	20.9	22.8	12.3	169	25.5	25.3	16.0
37	5.7	5.5	3.1	81	11.9	13.2	6.6	133	21.0	22.9	12.4	170	25.6	25.3	16.1
38	5.8	5.8	3.2	82	12.1	13.3	6.7	134	21.2	23.0	12.5	171	25.7	25.3	16.3
39	6.0	5.9	3.3	83	12.3	13.5	6.8	135	21.3	23.2	12.7	174	26.1	25.3	16.4
40	6.1	6.0	3.4	86	12.9	14.1	6.9	136	21.4	23.4	12.9	175	26.3	25.3	16.5
41	6.2	6.1	3.5	87	13.0	14.2	7.0	137	21.6	23.6	13.0	176	26.5	25.3	16.6
44	6.5	6.5	3.6	88	13.2	14.3	7.1	139	21.9	23.7	13.1	177	26.6	25.6	16.8
45	6.6	6.7	3.7	89	13.3	14.4	7.3	140	22.0	23.8	13.2	178	26.7	25.7	17.0
46	6.7	6.8	3.8	90	13.4	14.6	7.5	141	22.2	23.9	13.3	179	26.9	26.0	17.1
47	6.8	6.9	3.9	92	13.8	15.1	7.7	142	22.4	24.0	13.4	181	27.4	26.2	17.2

## Conclusions

This research has focused on determining the OTA of PV panels using simulations and experimental investigations. The experimental work consists of three PV panels installed at 28°, 30°, and 50° TAs. Measurements are recorded for 181-day. By analyzing the recoded data, it was found that the 28° TA has the best performance and the 50 TA has the worst performance. The SR of the 28° TA is higher than that of the 50^o^ TA by 59.3% and better than the TA of 30° by 4.5%. Beside the experimental work, the GTA and other 9 MHTs have been implemented to estimate the best values of the OTAs. Genetic algorithm, particle swarm, harmony search, ant colony, cuckoo search, bee colony, fire fly, grey wolf, and coronavirus disease optimizers are among the various implemented MHTs. The implemented MHTs calculated the OTA to be 28.445° for Suez city which agrees with the result obtained from experimental work. The OTA for several cities throughout the world are computed for comparison purposes. The results show that GTA and the 9 MHTs are both quite good at estimating the OTA.

## Data avaliability

The data that support the findings of this study are available from the corresponding author upon reasonable request.
